# Synthesis and Biological Assessment of Eucalyptin: Magic Methyl Effects

**DOI:** 10.3390/ijms26073391

**Published:** 2025-04-04

**Authors:** Kanta Fuchiyama, Yuka Yabuki, Yuzu Yamamoto, Ryuki Asakawa, Saki Matsumoto, Yuuka Ibayashi, Yuuki Furuyama, Kenji Ohgane, Shinji Kamisuki, Koichi Watashi, Makoto Matsubayashi, Kouji Kuramochi

**Affiliations:** 1 Department of Applied Biological Science, Faculty of Science and Technology, Tokyo University of Science, Noda 278-8510, Chiba, Japan6423511@ed.tus.ac.jp (Y.I.); y.furuyama@rs.tus.ac.jp (Y.F.); ohgane.kenji@ocha.ac.jp (K.O.); kwatashi@niid.go.jp (K.W.); 2 Department of Chemistry, Ochanomizu University, 2-1-1 Otsuka, Bunkyo-ku 112-8610, Tokyo, Japan; 3 School of Veterinary Medicine, Azabu University, 1-17-71 Fuchinobe, Chuo-ku, Sagamihara 252-5201, Kanagawa, Japan; kamisuki@azabu-u.ac.jp; 4 Center for Human and Animal Symbiosis Science, Azabu University, 1-17-71 Fuchinobe, Chuo-ku, Sagamihara 252-5201, Kanagawa, Japan; 5 Research Center for Drug and Vaccine Development, National Institute of Infectious Diseases, Shinjuku-ku 162-8640, Tokyo, Japan; 6 Department of Veterinary Immunology, Graduate School of Veterinary Sciences, Osaka Metropolitan University, Izumisano 598-8531, Osaka, Japan; matsubayashi@omu.ac.jp

**Keywords:** *C*-methylated flavonoid, eucalyptin, cytotoxicity, antibiofilm, magic methyl effect

## Abstract

A drastic alteration in the potency or efficacy of bioactive compounds due to the addition of a single methyl group is known as the magic methyl effect. This effect has been demonstrated in *O*-methylated flavonoids, which show differences in physical and chemical properties from those of unmethylated flavonoids—*O*-methylation converts the hydrophilic hydroxy group into a hydrophobic methoxy group. However, differences in the physical and chemical properties between *C*-methylated and unmethylated flavonoids are smaller than those for *O*-methylated flavonoids. Therefore, predicting the magic methyl effect in *C*-methylated flavonoids is challenging. Eucalyptin and 8-desmethyleucalyptin are *C*-methylated flavonoids isolated from the leaves of plants, such as *Eucalyptus* sp. and *Kalmia latifolia*. These compounds contain 5-hydroxy-7,4′-dimethoxyflavone as the core skeleton. Eucalyptin has two *C*-methyl groups at the C-6 and C-8 positions, whereas 8-desmethyleucalyptin has one *C*-methyl group at the C-6 position. In this study, we synthesized eucalyptin and assessed its biological activities. The C-8 methyl group caused significant alterations in cytotoxic and antibiofilm activities. Herein, we report the magic methyl effects in eucalyptin, providing a basis for further chemical and biological studies on *C*-methylated flavonoids.

## 1. Introduction

**Figure 1:**
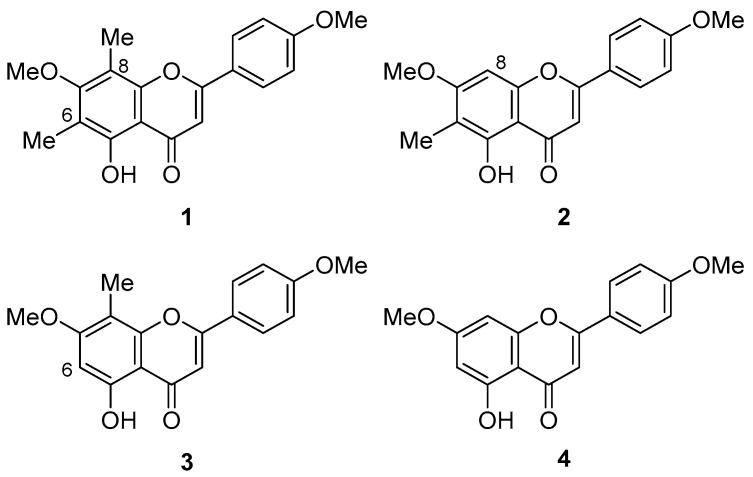
Structures of eucalyptin (**1**), 8-desmethyleucalyptin (**2**), 6-desmethyleucalyptin (**3**), and 5-hydroxy-7,4′-dimethoxyflavone (**4**).

Eucalyptin (**1**) and 8-desmethyleucalyptin (**2**) have been isolated from the leaves of various plants, such as *Eucalyptus* sp. and *Kalmia latifolia* (Figure 1) [1,2,3]. These compounds exhibit antibacterial activities against Gram-positive bacteria, such as *Staphylococcus aureus*, methicillin-resistant *S. aureus* (MRSA), *Bacillus cereus*, *Enterococcus faecalis*, *Alicyclobacillus acidoterrestris*, and *Cutibacterium acnes*, and antifungal activity against *Trichophyton mentagrophytes* [4]. They inhibit biofilm formation via both MRSA and methicillin-sensitive *S. aureus* [5]. In addition to their antimicrobial activities, these compounds reduce the activation of nuclear factor κB and activator protein 1 in THP-1-XBlue™-myeloid differentiation protein 2 (MD2)-CD14 cells—a human monocytic THP-1 cell line that stably expresses MD2 and CD14 [6]—indicating anti-inflammatory activity. They exhibit preferential cytotoxic activity (anti-austerity activity) against the PANC-1 human pancreatic cancer cell line in both nutrient-deprived and standard nutrient-rich (DMEM) medium [7].

**Scheme 1:**
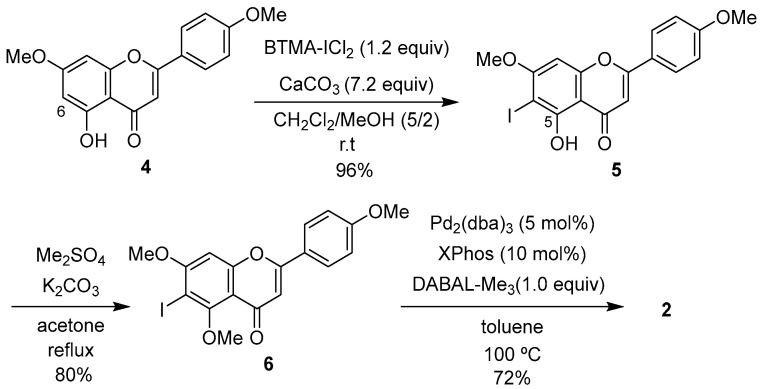
Our previous synthesis of **2**.

Only a few studies have reported the syntheses of **1** and **2** [8,9], including one report on the synthesis of **1** [9], to the best of our knowledge. Our group synthesized **2** and 6-desmethyleucalyptin (**3**) from 5-hydroxy-7,4′-dimethoxyflavone (**4**). The selective iodination of **4** at C-6 using benzyltrimethylammonium dichloroiodate (BTMA-ICl_2_) [10], methylation of the hydroxyl group at C-5 in **5**, and palladium-catalyzed methylation of **6** with the bis(trimethylaluminum)-1,4-diazabicyclo[2.2.2]octane adduct (DABAL-Me_3_) yielded **2** (Scheme 1) [11]. Palladium coupling was accompanied by demethylation at C-5. We observed that **2** exhibited cytotoxicity against human colon carcinoma HCT116 cells and normal diploid fibroblast MRC-5 cells. However, it did not exhibit cytotoxicity against T cell acute lymphoblastic leukemia Jurkat cells. Neither **3** nor **4** with a *C*-methyl group at C-6 exhibited cytotoxicity against HCT116, Jurkat, and MRC-5 cells. These results indicated that the C-6 methyl group is crucial for the cytotoxicity of **2**.

**Figure 2:**
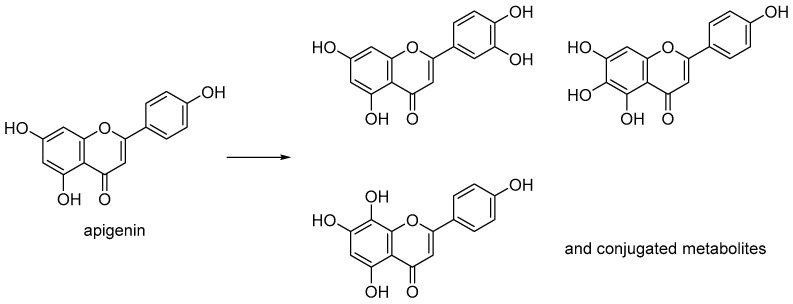
Metabolism of apigenin.

Methylation of flavonoids alters their biochemical properties, such as hydrophobicity and metabolic stability [12], thereby altering their biological activity. Flavonoid methylation enhances bioavailability and stability, promoting biological activities, such as anticancer, antiviral, and anti-inflammatory effects [13]. These modifications make methylated flavonoids promising candidates for pharmaceutical and nutraceutical applications. However, there are relatively few studies of the biological activities of *C*-methylated flavonoids compared with those of *O*-methylated flavonoids (methoxyflavonoids). Because of its hydrophobic methyl group, **1** was more hydrophobic than **2**. In vitro studies using rat liver models have demonstrated that apigenin, a structurally similar flavonoid, undergoes aromatic hydroxylation to form monohydroxylated metabolites and their conjugates, such as glucuronoconjugates and sulfoconjugates (Figure 2) [14]. Therefore, **1** may show lower susceptibility to metabolism than that of **2**. In summary, we expected biological activity to differ between **1** and **2**.

In this study, we synthesized **1** using the same approach as for **2**. We assessed biological activity and observed magic methyl effects [15] (i.e., the effects of the addition of a methyl group on pharmacological properties, particularly cytotoxicity and antibiofilm activity of **1**). The “magic methyl” effect has become a powerful strategy in drug discovery and drug development. Methylation at the C-6 position of **2** significantly altered its biological properties.

## 2. Results and Discussion

### 2.1. Synthesis of ***1***

**Scheme 2:**
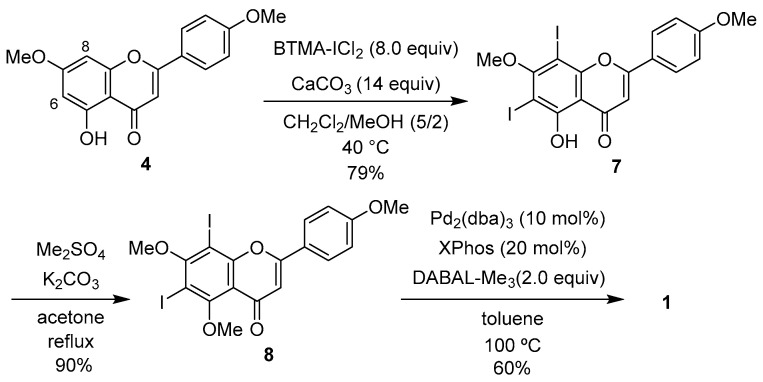
Synthesis of **1**.

The synthesis of **1** is illustrated in Scheme 2. Treatment of **4** with excess BTMA-ICl_2_ and CaCO_3_ at 40 °C yielded diiodide **7** as the single product in 79% yield. In contrast, an excess amount of *N*-iodosuccinimide provided an inseparable mixture of mono- and diiodides. The hydroxyl group in **7** was protected with methyl ether to form **8**. The palladium-catalyzed methylation of **8** using tris(dibenzylideneacetone)dipalladium {Pd_2_(dba)_3_},2-dicyclohexylphosphino-2′,4′,6′-triisopropylbiphenyl (XPhos) and DABAL-Me_3_ (2.0 equiv.) produced **1** in 60% yield. Therefore, we demonstrated that both **1** and **2** can be prepared from **3** using the same synthetic approach.

### 2.2. Cytotoxic Activity of ***1*** and ***2***

The cytotoxic activities of **1** against human cervical carcinoma HeLa, Jurkat, and MRC-5 cells were assessed using water-soluble tetrazolium salt (WST-8) assays [16] with **2** as a control. The concentrations of each compound required to reduce the cell viability by 50% (half-maximal inhibitory concentration) are summarized in Table 1. Both **1** and **2** exhibited cytotoxicity against HeLa cells. However, **1** exhibited cytotoxicity against Jurkat cells, whereas **2** exhibited no effect. This difference may be attributed to the hydrophobic properties and/or metabolic stability of **1**. Namely, the methyl group at the C-8 position in **1** could increase membrane permeability by increasing hydrophobicity. The methyl group at the C-8 position in **2** can protect the aromatic carbon at the C-8 position from metabolic hydroxylation. Additionally, **1** showed lower toxicity to normal MRC cells than that of **2**. Although the reason for this result remains unclear, **1** is considered a potential candidate for the development of chemotherapeutic agents.

### 2.3. Effects of ***1*** and ***2*** on the Biofilm Formation and Growth of Cutibacterium Acnes

**Figure 3:**
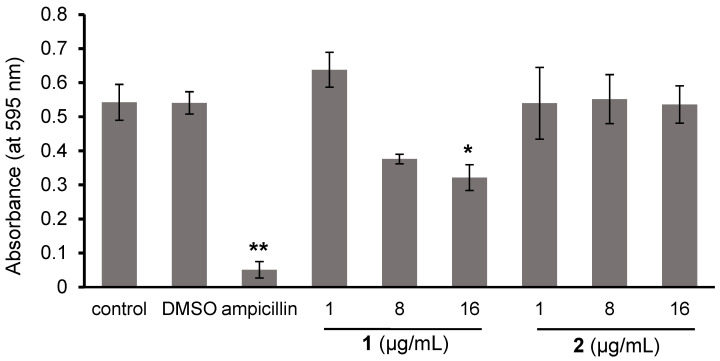
Effects of **1** and **2** on biofilm formation by *Cutibacterium acnes*. The biofilm biomass of *C. acnes* after incubation for 96 h under anaerobic conditions without (control) or with dimethyl sulfoxide (DMSO) (0.1%), ampicillin (150 μg/mL), and various concentrations of **1** and **2** was determined using crystal violet staining measured at an absorbance of 595 nm. Data are presented as the mean ± standard error of the mean of three independent experiments performed in triplicate. Statistical significance was assessed using Dunnett’s test; * *p* < 0.05 and ** *p* < 0.01, compared with that of the DMSO-treated control.

**Figure 4:**
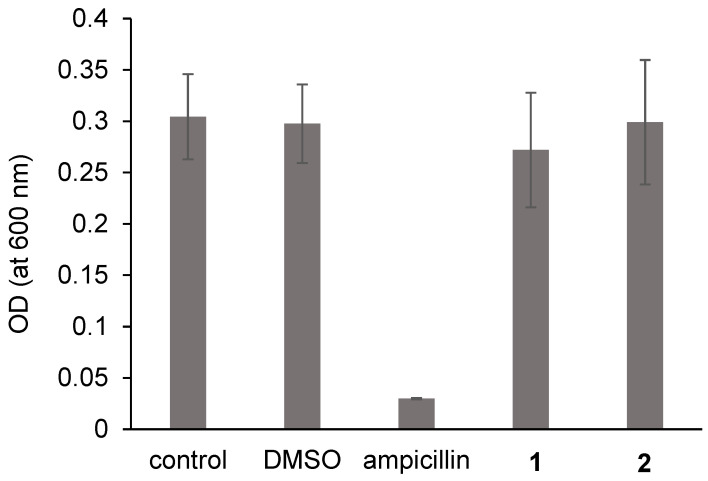
Effects of **1** and **2** on the growth of *Cutibacterium acnes*. Bacterial growth was measured as OD_600_ in the absence (control) or presence of dimethyl sulfoxide (0.1%), ampicillin (15 μg/mL), **1** (16 µg/mL), and **2** (16 µg/mL). Data are presented as the mean ± standard error of the mean of three independent experiments performed in triplicate.

The effects of **1** and **2** on biofilm formation by *C. acnes* were assessed using crystal violet staining (Figure 3). Compound **1** inhibited biofilm formation in a dose-dependent manner. At a concentration of 16 μg/mL, **1** reduced biofilm formation by 40% compared with that of the dimethyl sulfoxide (DMSO)-treated control, whereas **2** exhibited no inhibitory effect. Subsequently, the effects of **1** and **2** on *C. acnes* growth were assessed by measuring the optical density at 600 nm (OD_600_) (Figure 4). Neither **1** nor **2** inhibited growth at a concentration of 16 µg/mL. These results indicated that **1** inhibits biofilm formation without affecting bacterial growth and targets intracellular and/or extracellular biomolecules involved in biofilm formation. Because of the hydrophobic methyl group, **1** may be more prone to binding hydrophobic biomolecules.

## 3. Materials and Methods

### 3.1. General Procedure and Materials

All reactions sensitive to air or moisture were performed in an argon atmosphere under anhydrous conditions unless otherwise noted. The solvents and reagents were used without further purification unless otherwise noted. Analytical thin-layer chromatography (TLC) was performed using Silica gel 60 F_254_ plates (0.25 mm, normal phase, Merck, Rahway, NJ, USA). Normal phase flash column chromatography was performed using Silica gel 60 (particle size 40–63 μm; 230–400 mesh ASTM; SilicaFlash F60, SiliCycle Inc., Quebec, QC, Canada). Melting point (Mp) data were determined using a Shimadzu MM-2 instrument and uncorrected. The IR spectra were recorded on a Horiba FT-720 spectrometer using KBr pellets (solid). ^1^H and proton-decoupled ^13^C NMR spectra were recorded on a Bruker Avance 400 spectrometer (400 and 100 MHz, respectively) using chloroform-*d* (CDCl_3_) as the solvent. Chemical shift values are expressed in δ (ppm) relative to tetramethylsilane (TMS, δ 0.00 ppm for ^1^H NMR) or the residual solvent resonance (δ 77.0 ppm for ^13^C NMR). The following data were reported: chemical shift, multiplicity (s = singlet, d = doublet), coupling constants (*J*; Hz), and integration. The mass spectra were obtained using a JEOL high-resolution double-focusing mass spectrometer (JMS-700) with fast atom bombardment (FAB). Absorbance at 570 nm and OD600 were measured using a Multiskan GO microplate reader (Thermo Fisher Scientific, Waltham, MA, USA). CaCO_3_, K_2_CO_3_, Me_2_SO_4_, CH_2_Cl_2_, CHCl_3_, dimethyl sulfoxide (DMSO), yeast extract, glucose, NaOAc, agar, ampicillin, Dulbecco’s modified Eagle’s medium (DMEM), Roswell Park Memorial Institute (RPMI) 1640 medium, Cell Counting Reagent SF, crystal violet, and phosphate-buffered saline (PBS) were purchased from Nacalai Tesque (Kyoto, Japan). BTMA-ICl_2_, DABAL-Me_3_, Pd_2_(dba)_3_, and XPhos were purchased from Tokyo Chemical Industry Co., Ltd. (Tokyo, Japan). CH_3_OH, CDCl_3_, Na_2_S_2_O_3_, Na_2_SO_4_, NaCl, penicillin-streptomycin solution (×100), and Eagle’s Minimum Essential Medium (E-MEM) with l-glutamine and phenol red were obtained from FUJIFILM Wako Pure Chemical (Osaka, Japan). Fetal bovine serum (FBS) was purchased from BioWest (Nu-Aaillé, France). Difco Nutrient Broth was purchased from Becton Dickinson and Co. (Sparks, MD, USA). Compound **2** was previously prepared and used in this study [11]. All synthetic compounds were pure, as validated based on supplemental NMR spectra (Supplementary Figures S1–S8).

### 3.2. Chemistry

#### 3.2.1. Synthesis of 5-Hydroxy-6,8-diiodo-7-methoxy-2-(4-methoxyphenyl)-4H-chromen-4-one (**7**)

CaCO_3_ (2.35 g, 23.5 mmol) followed by BTMA-ICl_2_ (4.67 g, 13.4 mmol) was added to a solution of **3** (500 mg, 1.68 mmol) in CH_2_Cl_2_/CH_3_OH (5/2, 31.5 mL). The mixture was stirred at 40 °C for 47.5 h. The reaction was quenched by the addition of a saturated aqueous Na_2_S_2_O_3_ solution and diluted with CHCl_3_. After the layers separated, the aqueous layer was extracted with CHCl_3_. The combined organic layers were washed with H_2_O and brine, dried over Na_2_SO_4_, and then concentrated. The residue was purified by trituration with EtOH to yield compound **7** (730 mg, 1.32 mmol, 79%) as a yellow solid. Mp = 250–251 °C; IR (KBr) ν_max_ = 3442, 3084, 3005, 2972, 2939, 2837, 1645, 1606, 1572, 1508, 1456, 1427 cm^−1^; ^1^H NMR (400 MHz, CDCl_3_) δ 14.06 (s, 1H), 8.03 (d, *J* = 8.8 Hz, 2H), 7.07 (d, *J* = 9.2 Hz, 2H), 6.74 (s, 1H), 3.98 (s, 3H), 3.92 (s, 3H); ^13^C NMR (100 MHz, CDCl_3_) δ 181.8, 165.2, 164.6, 163.3, 162.2, 156.3, 128.8, 122.7, 114.8, 108.2, 103.7, 75.9, 67.5, 61.2, 55.6; HRMS (ESI+) *m*/*z* calcd for C_17_H_13_I_2_O_5_ ([M + H]^+^) 550.8847, found 550.8850.

#### 3.2.2. Synthesis of 6,8-Diiodo-5,7-dimethoxy-2-(4-methoxyphenyl)-4H-chromen-4-one (**8**)

K_2_CO_3_ (654 mg, 4.73 mmol), followed by Me_2_SO_4_ (455 μL, 4.69 mmol), was added to a solution of **7** (258 mg, 0.469 mmol) in acetone (40 mL) at room temperature. The mixture was refluxed for 40.5 h. The reaction was quenched by adding H_2_O and diluted with CHCl_3_. After the layers separated, the aqueous layer was extracted with CHCl_3_. The combined organic layers were washed with brine, dried over Na_2_SO_4_, and concentrated. The residue was purified using silica gel column chromatography (toluene/acetone = 100/1) to yield compound **8** (238 mg, 0.421 mmol, 90%) as a yellow solid. Mp = 222–223 °C; IR (KBr) ν_max_ = 3448, 2939, 2846, 1647, 1608, 1560, 1512, 1454, 1423, 1402 cm^−1^; ^1^H NMR (400 MHz, CDCl_3_) δ 8.00 (d, *J* = 8.8 Hz, 2H), 7.03 (d, *J* = 8.8 Hz, 2H), 6.68 (s, 1H), 3.97 (s, 3H), 3.95 (s, 3H), 3.90 (s, 3H); ^13^C NMR (100 MHz, CDCl_3_) δ 175.7, 163.5, 162.7, 162.1, 161.1, 157.7, 128.4, 123.0, 116.1, 114.6, 106.6, 87.82, 62.22, 61.08, 55.55; HRMS (ESI+) *m*/*z* calcd for C_18_H_15_I_2_O_5_ ([M + H]^+^) 564.9003, found 564.9005.

#### 3.2.3. Synthesis of Eucalyptin (**1**)

Pd_2_(dba)_3_ (16.8 mg, 18.3 μmol), XPhos (17.4 mg, 36.5 μmol) and DABAL-Me_3_ (93.7 mg, 0.366 mmol) were added to a solution of **8** (103 mg, 0.183 mmol) in toluene (5 mL). The mixture was stirred at 100 °C for 3 h. The reaction was quenched by adding 1 M aqueous HCl, and the resultant mixture was diluted with CHCl_3_. After the layers separated, the aqueous layer was extracted with CHCl_3_. The combined organic layers were washed with brine, dried over Na_2_SO_4_, and concentrated. The residue was purified using silica gel column chromatography (toluene) to yield **1** (35.8 mg, 0.110 mmol, 60%) as a yellow solid. Mp = 188–190 °C; IR (KBr) ν_max_ = 3076, 2924, 2837, 1657, 1608, 1587, 1510, 1471, 1442, 1427 cm^−1^; ^1^H NMR (400 MHz, CDCl_3_) δ 12.87 (s, H), 7.87 (d, *J* = 9.2 Hz, 2H), 7.03 (d, *J* = 9.2 Hz, 2H), 6.61 (s, 1H), 3.90 (s, 3H), 3.80 (s, 3H), 2.39 (s, 3H), 2.21 (s, 3H); ^13^C NMR (100 MHz, CDCl_3_) δ 183.3, 163.9, 162.6, 157.3, 153.0, 128.0, 123.9, 114.6, 114.1, 108.8, 107.4, 104.1, 60.52, 55.55, 8.58, 8.29; HRMS (ESI+) *m*/*z* calcd for C_19_H_19_O_5_ ([M + H]^+^) 327.1227, found 327.1225.

### 3.3. Biology

#### 3.3.1. Cell Culture

HeLa cells were purchased from the RIKEN Bioresource Center (RIKEN BRC, Tsukuba, Japan). Jurkat cells were purchased from the Resource Center for Biomedical Research, Institute of Development, Aging and Cancer, Tohoku University (Sendai, Japan). MRC-5 cells were purchased from the National Institute of Biomedical Innovation, Health, and Nutrition (Ibaraki, Osaka, Japan). HeLa cells were cultured at 37 °C with 5% CO_2_ in D-MEM (Low Glucose) with l-Glutamine and Phenol Red medium supplemented with 10% FBS, 100 units/mL penicillin, and 100 μg/mL streptomycin. Jurkat cells were cultured at 37 °C with 5% CO_2_ in RPMI medium supplemented with 10% FBS, 100 units/mL penicillin, and 100 μg/mL streptomycin. MRC-5 cells were cultured in E-MEM with l-glutamine and phenol red supplemented with 10% FBS, 100 units/mL penicillin, and 100 μg/mL streptomycin.

#### 3.3.2. Cytotoxic Evaluation

Cell growth was evaluated using Cell Count Reagent SF according to the manufacturer’s instructions based on the WST-8 [2-(2-methoxy-4-nitrophenyl)-3-(4-nitrophenyl)-5-(2,4-disulfophenyl)-2*H*-tetrazolium, monosodium salt] assay. For the assay, the adherent HeLa and MRC-5 cells were cultured in a 96-well plate (SARSTEDT, Nümbrecht, Germany), with each well containing 2000 cells in a total volume of 100 μL. The Jurkat cells were cultured in a 96-well plate (AS ONE, Osaka, Japan), with each well containing 5000 cells in a total volume of 100 μL. The concentration of DMSO used in the cell culture was 1.0% (*v*/*v*). The plates also included blank wells (0 cells/100 μL) and control wells (2000 cells/100 μL for adherent cells and 5000 cells/100 μL for Jurkat cells). HeLa and MRC-5 cells were pre-incubated for 24 h before exposure to the test compounds. Jurkat cells were exposed to the test compounds without preincubation. HeLa and Jurkat cells were incubated with various concentrations of each compound for 48 h, and MRC-5 cells were incubated with various concentrations of each compound for 72 h. At the end of incubation, 10 μL of the WST-8 solution was then added, and the resulting mixture was incubated for 2 h at 37 °C. Absorbance was measured at 450 nm using a 96-well plate reader. Cell growth inhibition was calculated as the ratio of the absorbance of the sample to that of the control (Supplementary Figure S9).

#### 3.3.3. Preparation of Modified Reinforced Clostridial Medium (RCM)

Modified RCM liquid broth was prepared by dissolving 20.0 g of Difco Nutrient Broth, 3.0 g of yeast extract, 0.5 g of glucose, 5.0 g of NaCl, 3.0 g of NaOAc, and 0.5 g of agar in 1 L of deionized distilled water (DDW). The modified RCM agar was prepared by adding agar (0.3 g) to the liquid broth. The medium was then autoclaved at 121 °C for 15 min and cooled. Of note, *C. acnes* could not be cultured effectively in common RCM [17].

#### 3.3.4. Bacterial Strain and Growth Conditions

The strain *C. acnes* subsp. *acnes* JCM 6425^T^ was purchased from RIKEN BioResource Research Center Microbe Division/Japan Collection of Microorganisms (Tsukuba, Ibaraki, Japan). The strain was cultured on the modified RCM agar at 37 °C under anaerobic conditions. A single colony of *C. acnes* was inoculated into modified RCM broth (10 mL). The cells were grown anaerobically at 37 °C and resuspended in the modified RCM liquid broth to an appropriate optical density at 600 nm (OD_600_).

#### 3.3.5. Effects of **1** and **2** on Biofilm Formation of *C. acnes*

Biofilm formation was evaluated by crystal violet staining [18]. Crystal violet solution (0.1%) in methanol and DDW (1:4) was prepared. *C. acnes* with an OD_600_ of >3.0 was diluted 1:4 with modified RCM liquid broth. The *C. acnes* suspension was distributed in a 96-well plate (AS ONE, Osaka, Japan) and treated with ampicillin (150 µg/mL) or tested compounds (1, 8, and 16 µg/mL). A 0.1% DMSO-treated sample was used as a control. The mixture (200 μL) was incubated at 37 °C under anaerobic conditions for four days. Planktonic cells were removed, and the wells were washed twice with PBS. The biofilms were fixed with methanol (200 μL) for 15 min. After fixation, the methanol was removed and the plates were dried and stained with 0.1% crystal violet solution (150 μL) for 20 min. The plates were rinsed twice with PBS. Crystal violet was extracted from the biofilm using 95% ethanol (200 μL), and absorbance was measured at 595 nm using a microplate reader.

#### 3.3.6. Effects of **1** and **2** on the Growth of *C. acnes*

*C. acnes* was diluted with the modified RCM liquid broth to obtain a turbidity of 0.75 ± 0.25 McFarland turbidity standard. The *C. acnes* suspension was distributed in a 96-well plate (AZ ONE) and treated with ampicillin (15 µg/mL) or tested compounds (16 µg/mL). A 0.1% DMSO-treated sample was used as a control. The mixture (100 μL) was incubated at 37 °C under anaerobic conditions for 24 h. Bacterial growth was estimated by measuring the OD_600_ using a microplate reader.

## 4. Conclusions

**Figure 5:**
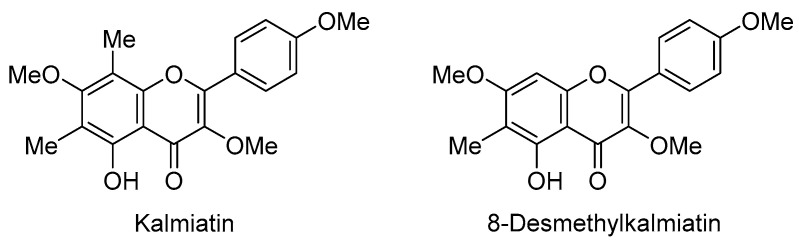
Structures of kalmiatin and 8-desmethylkalmiatin.

In this study, we report the synthesis, cytotoxicity against human cancer and normal cells, and antibiofilm activity of **1**. Compound **1** was synthesized using the same synthetic strategy as **2**—involving the iodation of **3** and palladium-catalyzed methylation of the corresponding iodides. This synthetic approach benefits from the ability to synthesize both monomethyl and dimethyl flavonoids from common non-methylated flavonoids. This strategy can be applicable to the synthesis of other *C*-flavonoids, such as kalmiatin and 8-desmethylkamiatin (Figure 5) [19].

Magic methyl effects were observed during the biological assessment of **1**. The addition of a methyl group at the C-8 position of **2** enhanced the cytotoxicity and antibiofilm activity significantly.

This study highlights the significance of both synthetic and biological studies on *C*-methylated flavonoids. The results offer valuable insights into the design and chemical modification of flavonoids. Ongoing research into the origin of magic methyl effects will be reported in the future.

## Figures and Tables

**Table 1:** Cytotoxicity of **1** and **2** against HeLa, Jurkat, and MRC-5 cell lines ^1^.

	IC_50_ (µM)		
Compounds	HeLa	Jurkat	MRC-5
**1**	18.3 ± 6.4	4.4 ± 1.2	>100
**2**	8.3 ± 0.5	>100	23.1 ± 6.0

^1^ Half-maximal inhibitory concentration (IC_50_) for cell viability after treatment with various concentrations of each compound, determined using the WST-8 assay. Three independent experiments were performed in triplicate. IC_50_ values are expressed as the mean ± standard error of the mean of three independent experiments (*n* = 3).

## Data Availability

Data are contained within the article.

## Supplementary Materials

The following supporting information can be downloaded at: https://www.mdpi.com/article/10.3390/ijms26073391/s1.
