# Supplementary material for: Synthesis and Biological Assessment of Eucalyptin: Magic Methyl Effects
Source: Int J Mol Sci. 2025 Apr 4;26(7):3391. doi: 10.3390/ijms26073391 (PMC11989289; doi:10.3390/ijms26073391)
Supplement: Supplementary file 1 [file ijms-26-03391-s001.zip › ijms-3509822-supplementary.pdf]

Supplementary Material

Synthesis and Biological Assessment of Eucalyptin: Magic Methyl Effects

Kanta Fuchiyama,[1] Yuka Yabuki,[1] Yuzu Yamamoto,[1] Ryuki Asakawa,[1] Saki Matsumoto,[1] Yuuka Ibayashi,[1] Yuuki Furuyama,[1] Kenji Ohgane,[1,2] Shinji Kamisuki,[3,4] Koichi Watashi,[1,5] Makoto Matsubayashi,[6] Kouji Kuramochi[1,*]

1. Department of Applied Biological Science, Faculty of Science and Technology, Tokyo University of Science, Noda, Chiba, 278-8510, Japan
2. Department of Chemistry, Ochanomizu University, 2-1-1 Otsuka, Bunkyo-ku, Tokyo, Japan
3. School of Veterinary Medicine, Azabu University, 1-17-71 Fuchinobe, Chuo-ku, Sagamihara, Kanagawa, 252-5201, Japan
4. Center for Human and Animal Symbiosis Science, Azabu University, 1-17-71 Fuchinobe, Chuo-ku, Sagamihara, Kanagawa, 252-5201, Japan
5. Research Center for Drug and Vaccine Development, National Institute of Infectious Diseases, Shinjuku-ku, Tokyo 162–8640, Japan
6. Department of Veterinary Immunology, Graduate School of Veterinary Sciences, Osaka Metropolitan University, Izumisano, Osaka, 598-8531, Japan

Index

Figure S1. ^{1}H NMR spectrum (400 MHz, $CDCl_3$, TMS) of compound **7** SI-2

Figure S2. ^{13}C NMR spectrum (100 MHz, $CDCl_3$) of compound **7** SI-3

Figure S3. ^{1}H NMR spectrum (400 MHz, $CDCl_3$, TMS) of compound **8** SI-4

Figure S4. ^{13}C NMR spectrum (100 MHz, $CDCl_3$) of compound **8** SI-5

Figure S5. ^{1}H NMR spectrum (400 MHz, $CDCl_3$, TMS) of compound **1** SI-6

Figure S6. ^{13}C NMR spectrum (100 MHz, $CDCl_3$) of compound **1** SI-7

Figure S7. ^{1}H NMR spectrum (400 MHz, $CDCl_3$, TMS) of compound **2** SI-8

Figure S8. ^{1}H NMR spectrum (400 MHz, $CDCl_3$, TMS) of compound **2** SI-9

Figure S9. Dose-response curves of **1** and **2** for the cytotoxicity assays SI-10

Figure S1. ^{1}H NMR spectrum (400 MHz, $CDCl_3$, TMS) of compound **7**

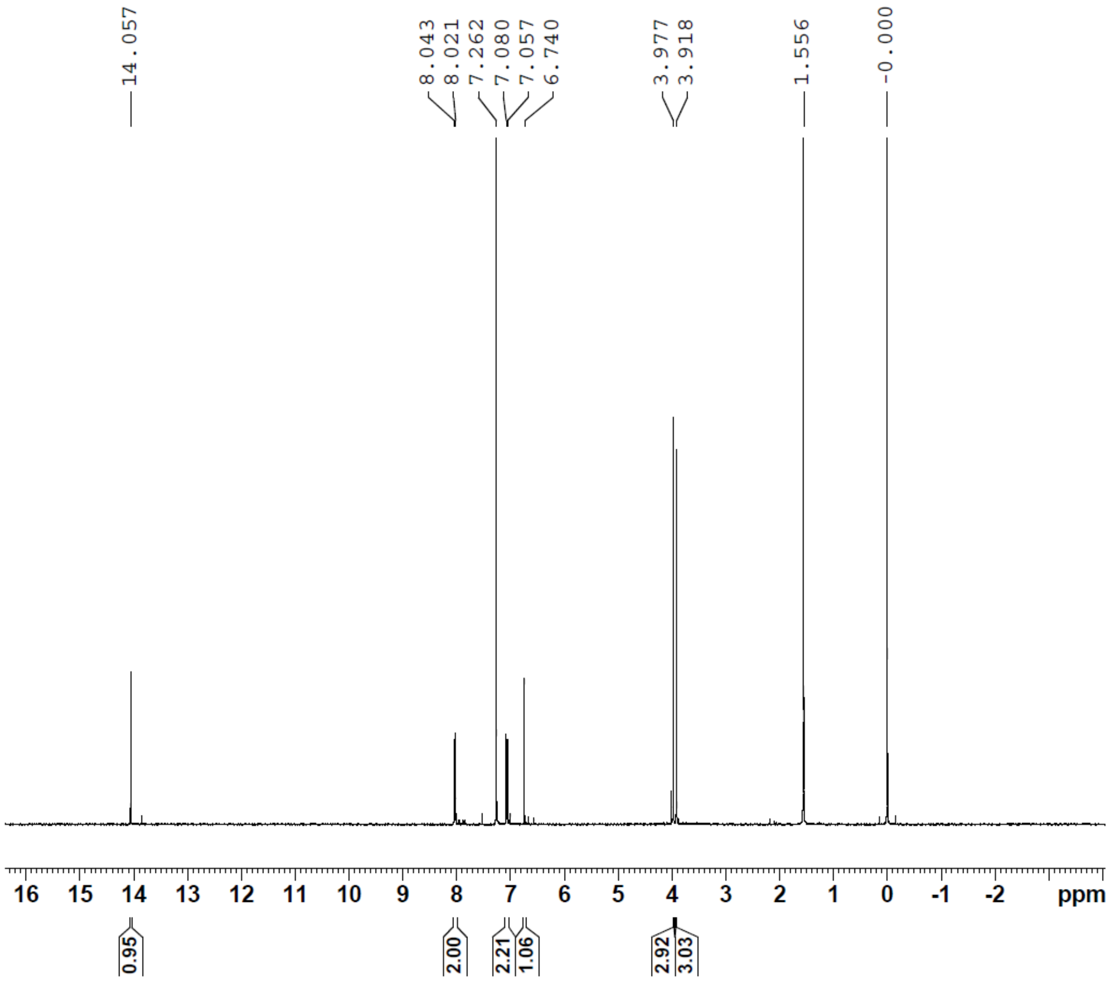

Figure S2. ^{13}C NMR spectrum (100 MHz, $CDCl_3$) of compound **7**

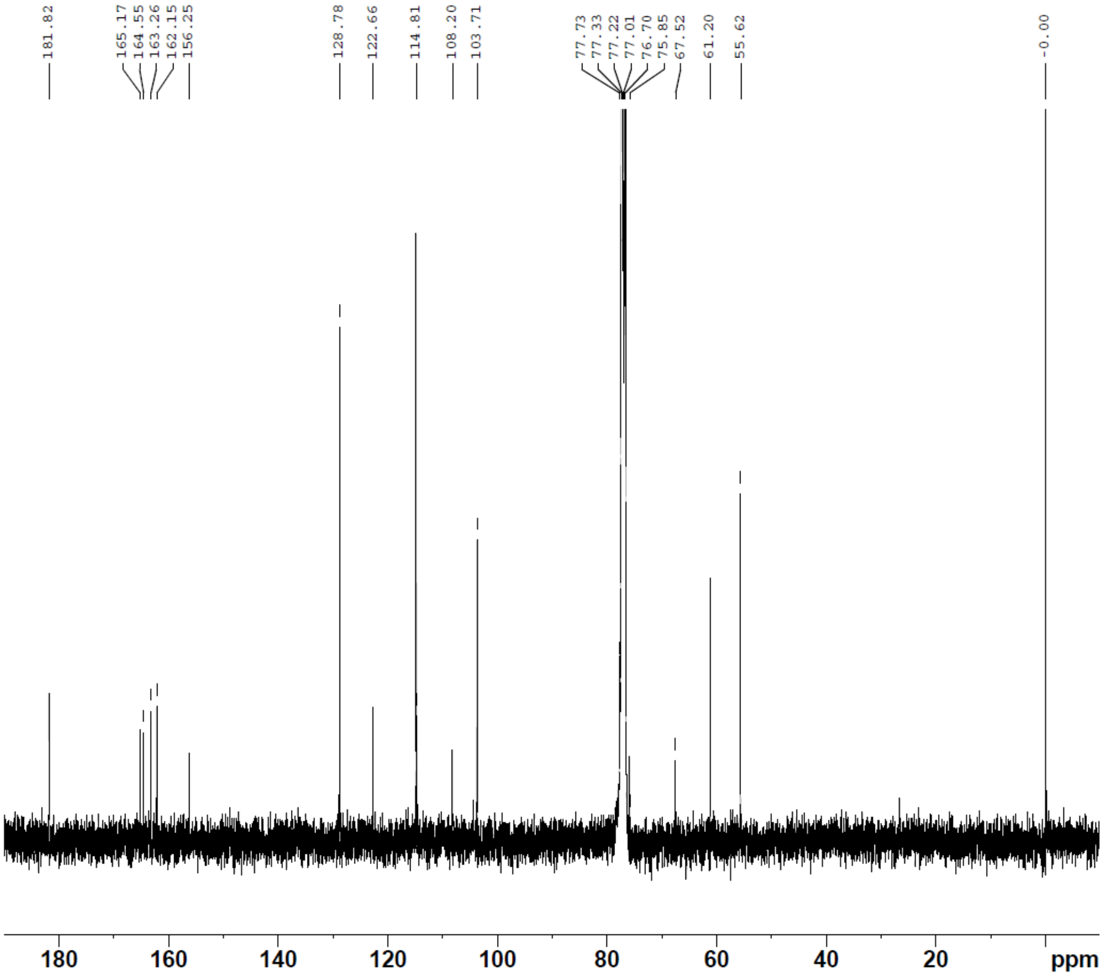

Figure S3. ^{1}H NMR spectrum (400 MHz, $CDCl_3$, TMS) of compound **8**

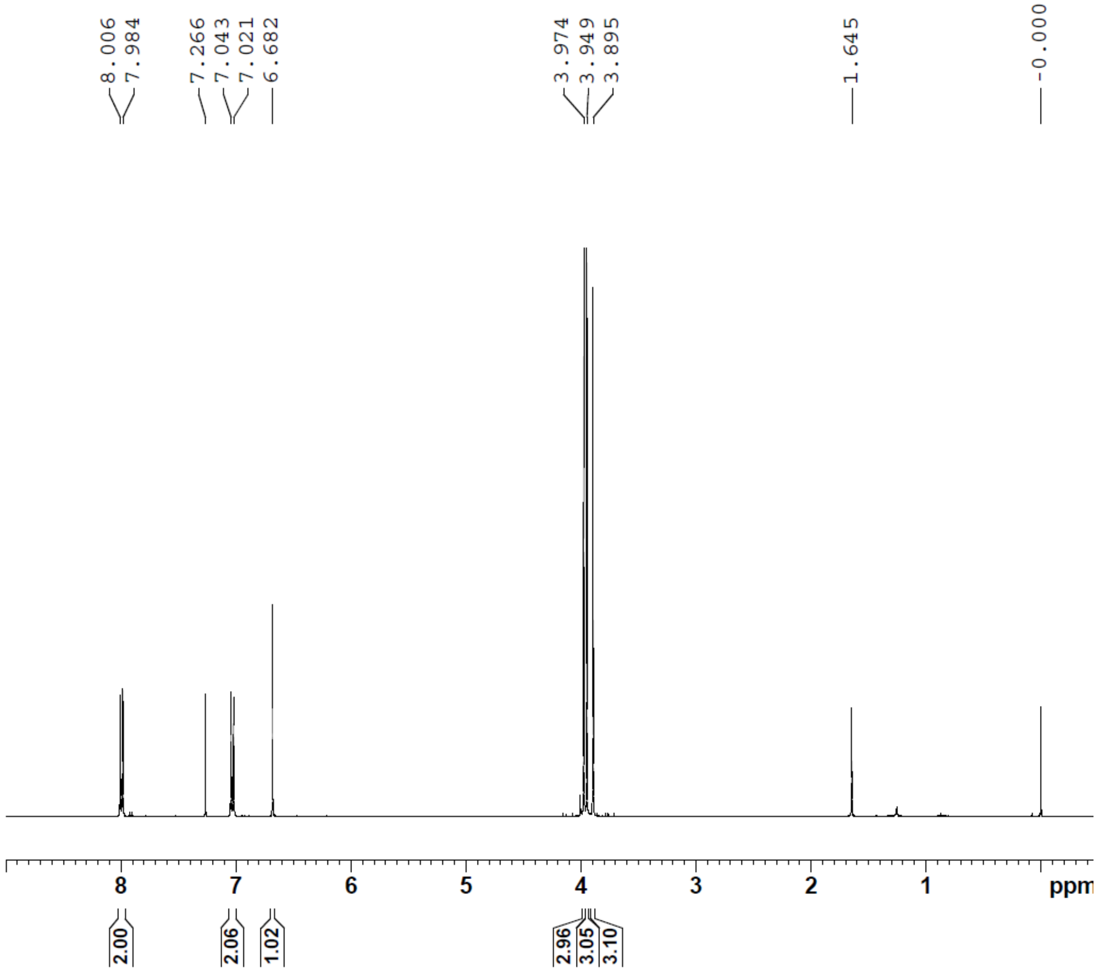

Figure S4. ^{13}C NMR spectrum (100 MHz, $CDCl_3$) of compound **8**

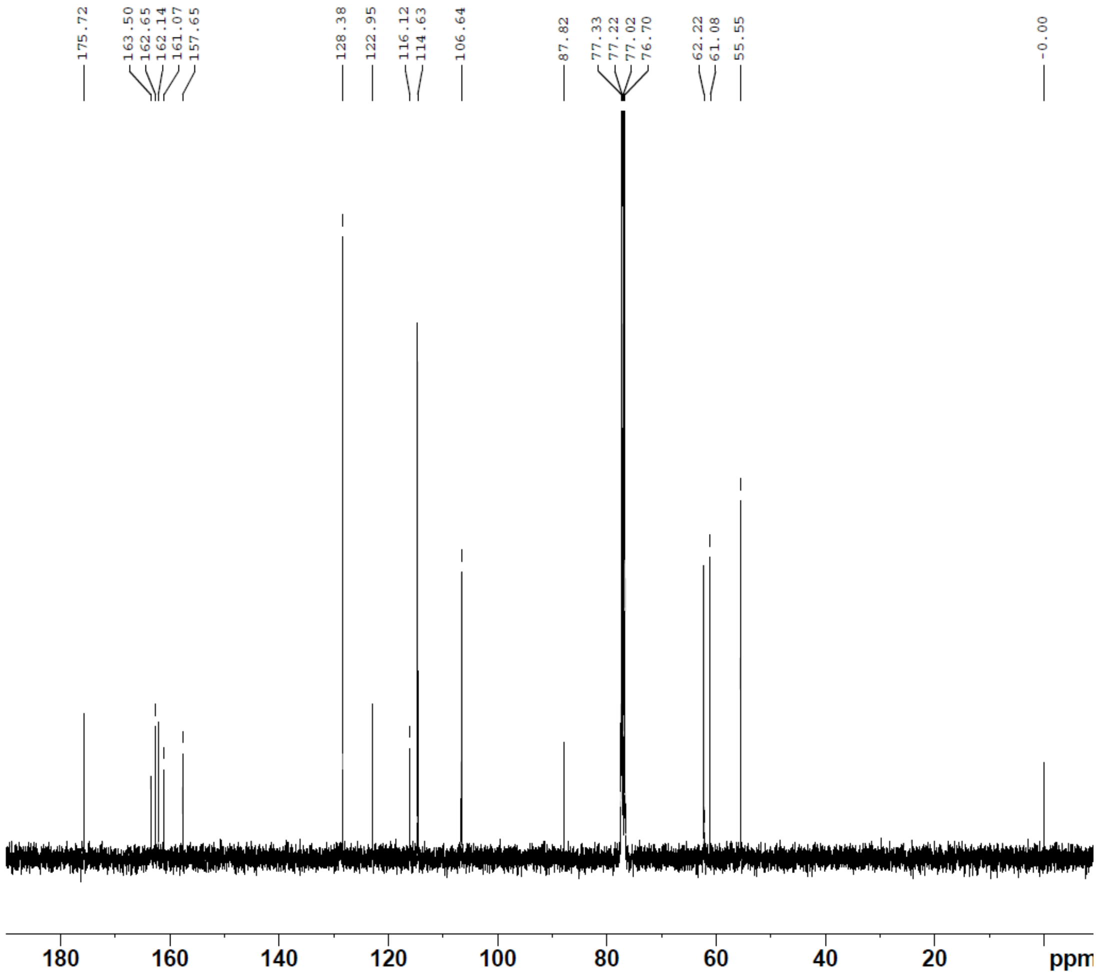

Figure S5. ^{1}H NMR spectrum (400 MHz, $CDCl_3$, TMS) of compound **1**

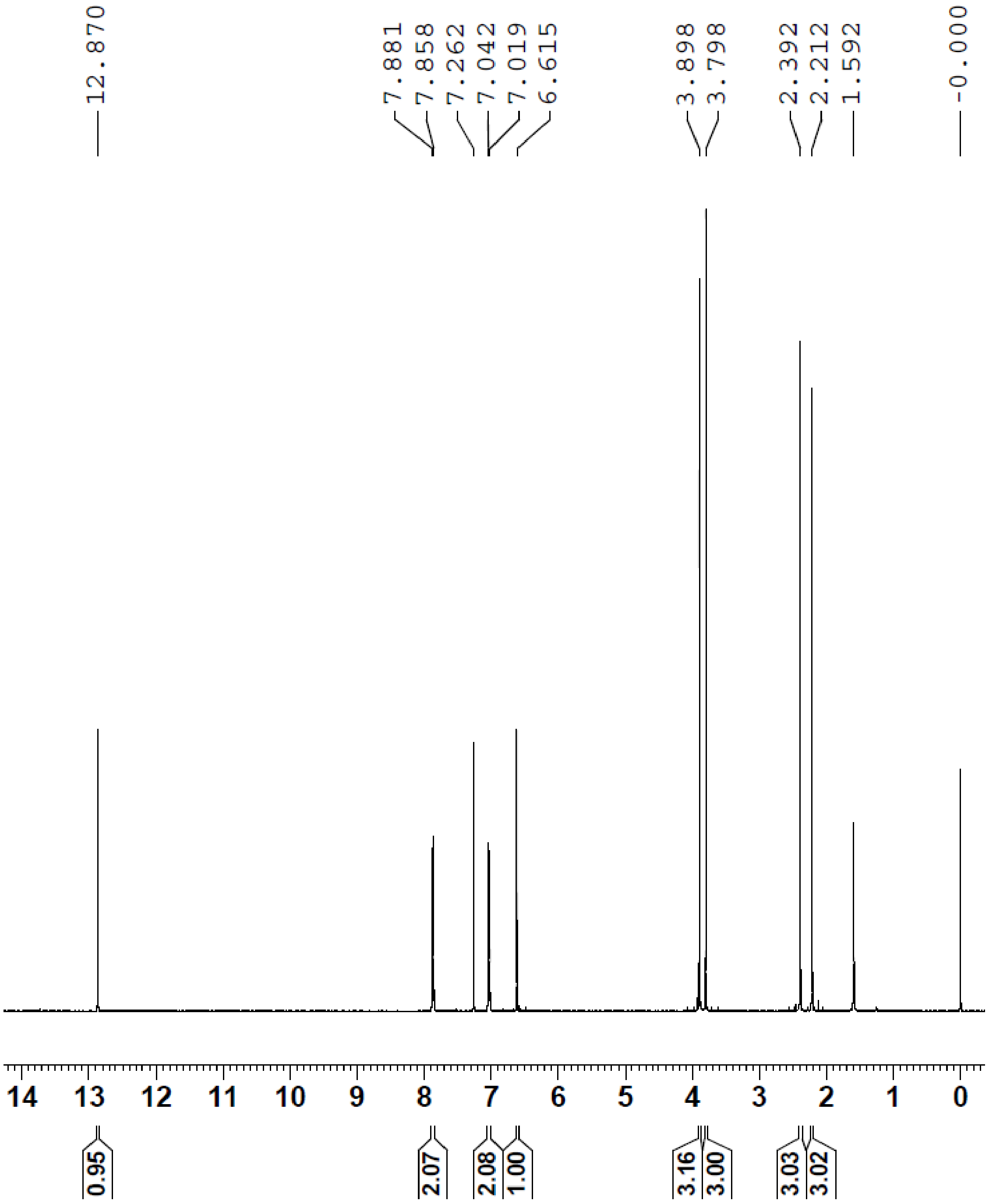

Figure S6. ^{13}C NMR spectrum (100 MHz, $CDCl_3$) of compound **1**

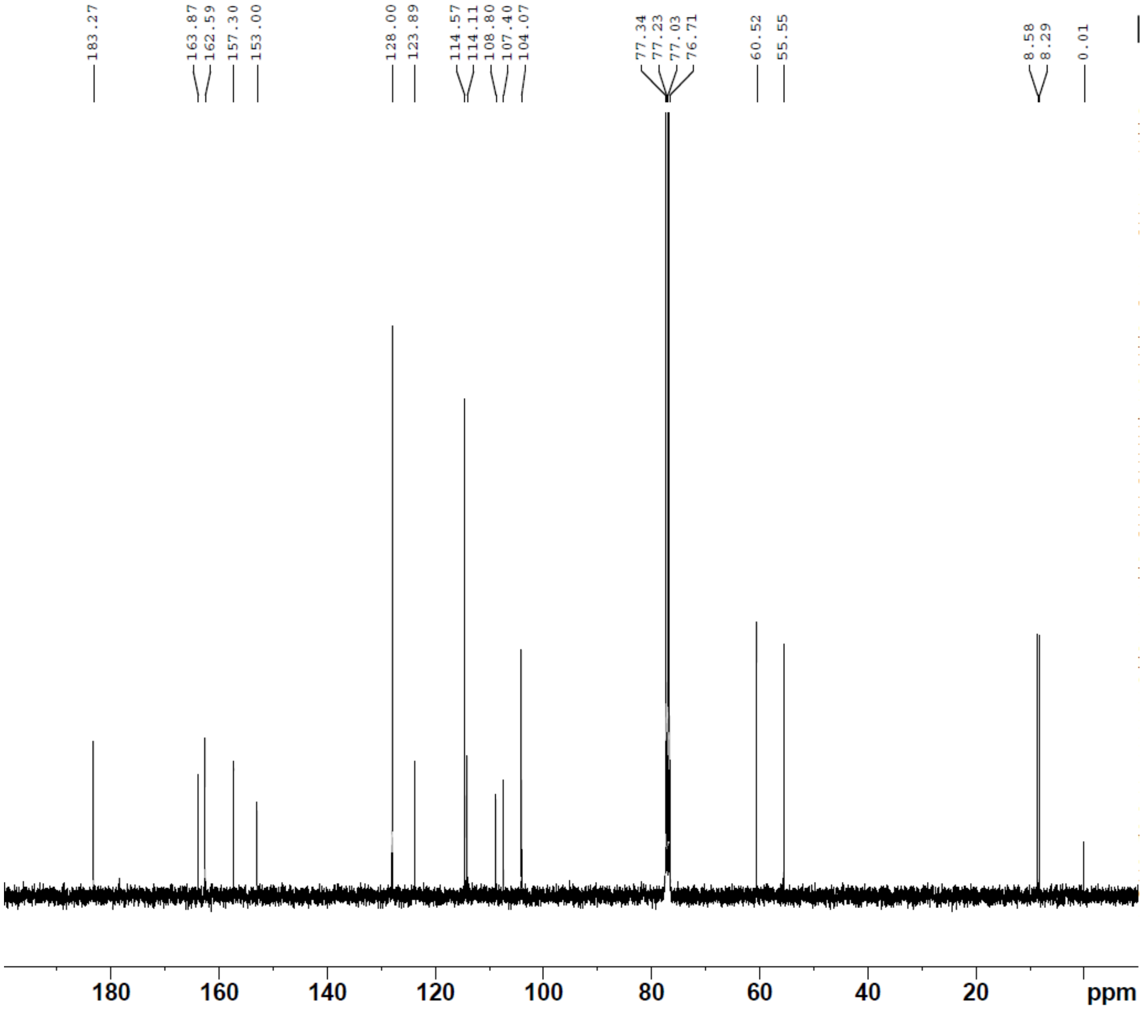

Figure S8. ^{1}H NMR spectrum (400 MHz, $CDCl_3$, TMS) of compound **2**

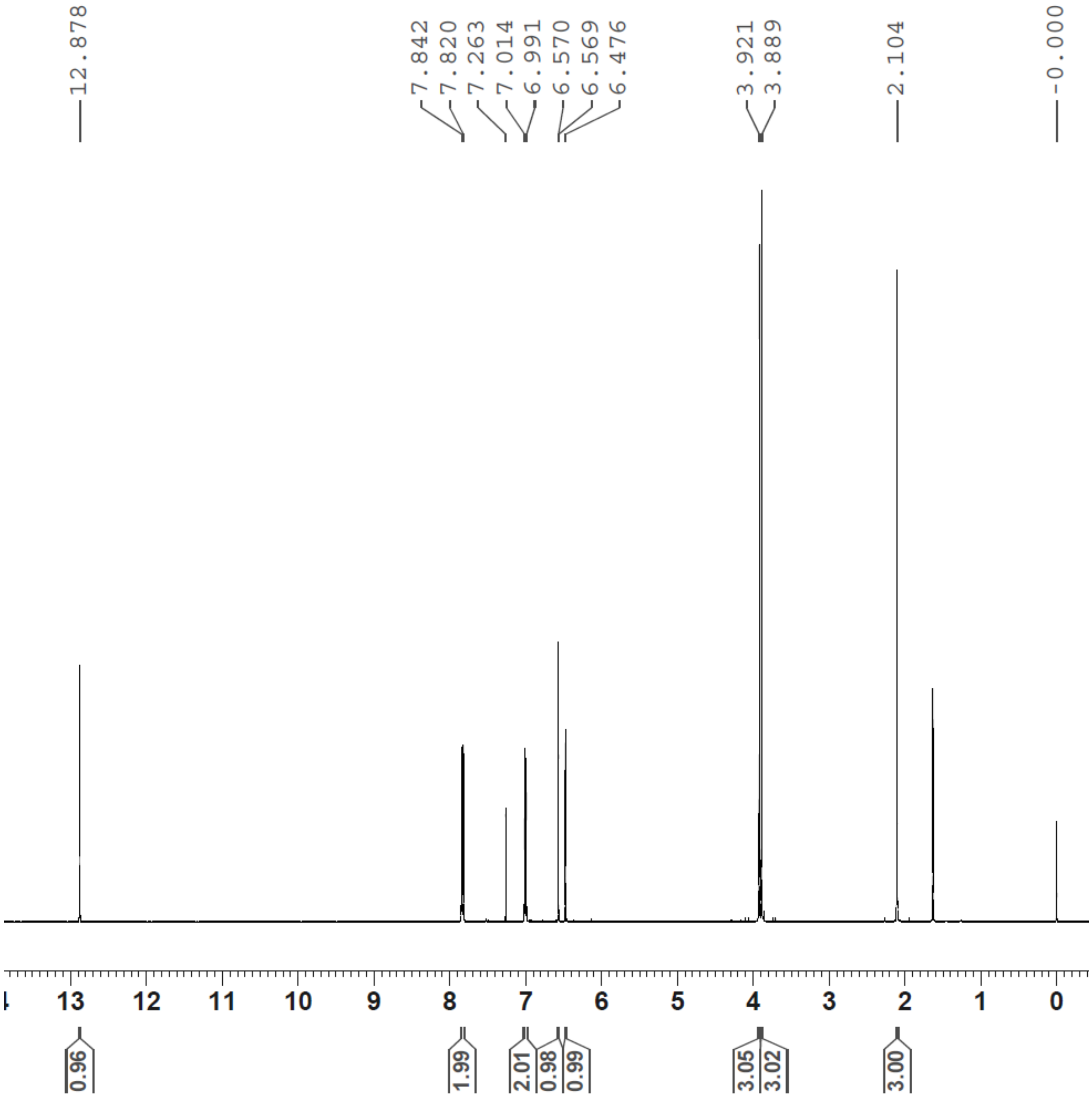

Figure S8. ^{13}C NMR spectrum (100 MHz, $CDCl_3$) of compound **2**

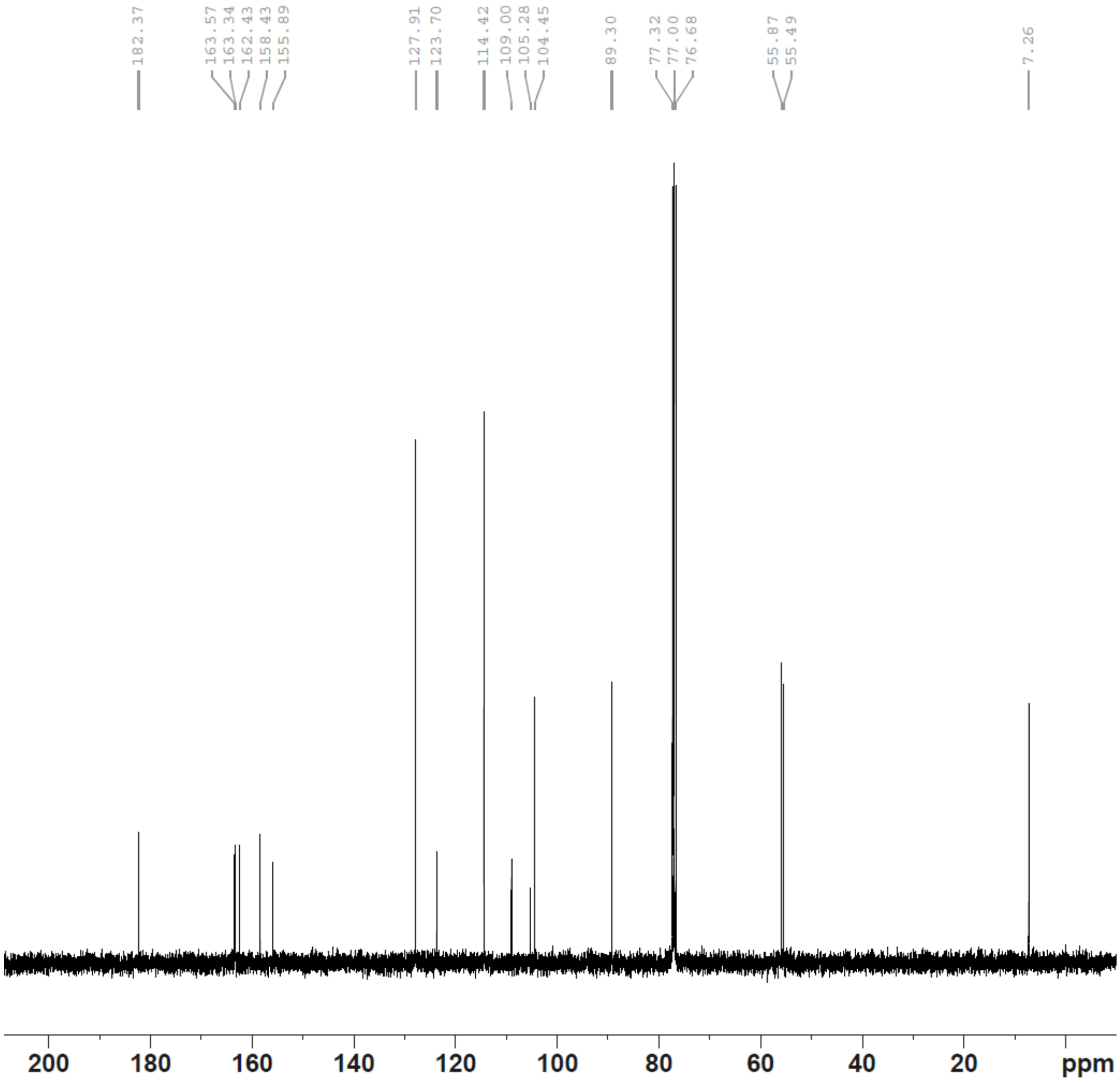

Figure S9. Dose-response curves of **1** and **2** for the cytotoxicity assays[a]

(A) HeLa cells

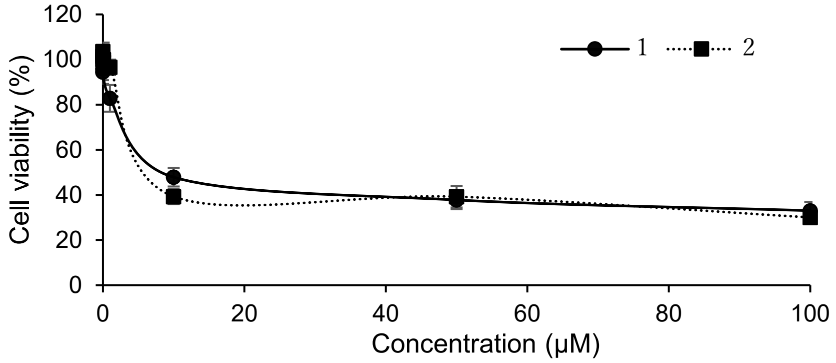

(B) Jurkat cells

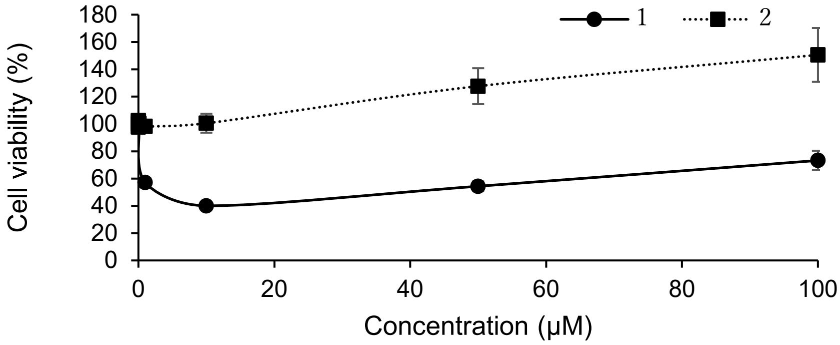

(C) MRC-5 cells

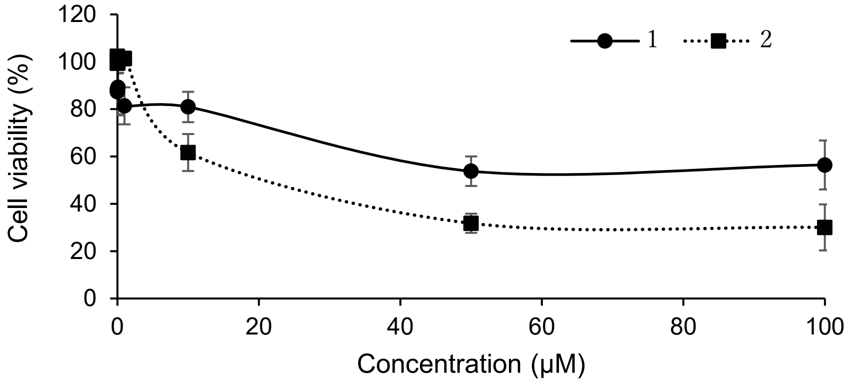

[a]Cell viability was determined by WST-8 assay. For the assay, the adherent HeLa and MRC-5 cells were cultured in a 96-well plate with each well containing 2000 cells. Jurkat cells were cultured in a 96-well plate with each well containing 5000 cells. The plates also included blank wells (0 cells/100 μL) and control wells (2000 cells/100 μL for the adherent cells and 5000 cells/100 μL for Jurkat cells). HeLa and MRC-5 cells were preincubated for 24 h before exposure to the test compounds. Jurkat cells were exposed to the test compounds without preincubation. HeLa and Jurkat cells were incubated with various concentrations

of each compound for 48 h. MRC-5 cells were incubated with various concentrations of each compound for 72 h. Data are shown as the mean ± SEM of three independent experiments.
